# Juvenile Hormone Biosynthesis Gene Expression in the *corpora allata* of Honey Bee (*Apis mellifera* L.) Female Castes

**DOI:** 10.1371/journal.pone.0086923

**Published:** 2014-01-29

**Authors:** Ana Durvalina Bomtorin, Aline Mackert, Gustavo Conrado Couto Rosa, Livia Maria Moda, Juliana Ramos Martins, Márcia Maria Gentile Bitondi, Klaus Hartfelder, Zilá Luz Paulino Simões

**Affiliations:** 1 Departamento de Genética, Faculdade de Medicina de Ribeirão Preto, Universidade de São Paulo, Ribeirão Preto, Brazil; 2 Departamento de Ciências do Ambiente, Universidade Federal de Mato Grosso do Sul, Campus do Pantanal, Corumbá, Brazil; 3 Departamento de Biologia, Faculdade de Filosofia Ciências e Letras de Ribeirão Preto, Universidade de São Paulo, Ribeirão Preto, Brazil; 4 Departamento de Biologia Celular e Molecular e Bioagentes Patogênicos, Faculdade de Medicina de Ribeirão Preto, Universidade de São Paulo, Ribeirão Preto, Brazil; University of Freiburg, Germany

## Abstract

Juvenile hormone (JH) controls key events in the honey bee life cycle, *viz.* caste development and age polyethism. We quantified transcript abundance of 24 genes involved in the JH biosynthetic pathway in the *corpora allata-corpora cardiaca* (CA-CC) complex. The expression of six of these genes showing relatively high transcript abundance was contrasted with CA size, hemolymph JH titer, as well as JH degradation rates and JH esterase (*jhe*) transcript levels. Gene expression did not match the contrasting JH titers in queen and worker fourth instar larvae, but *jhe* transcript abundance and JH degradation rates were significantly lower in queen larvae. Consequently, transcriptional control of JHE is of importance in regulating larval JH titers and caste development. In contrast, the same analyses applied to adult worker bees allowed us inferring that the high JH levels in foragers are due to increased JH synthesis. Upon RNAi-mediated silencing of the methyl farnesoate epoxidase gene (*mfe*) encoding the enzyme that catalyzes methyl farnesoate-to-JH conversion, the JH titer was decreased, thus corroborating that JH titer regulation in adult honey bees depends on this final JH biosynthesis step. The molecular pathway differences underlying JH titer regulation in larval caste development versus adult age polyethism lead us to propose that *mfe* and *jhe* genes be assayed when addressing questions on the role(s) of JH in social evolution.

## Introduction

Juvenile Hormone (JH), synthesized by the *corpora allata* (CA), a small pair of glands located in the retrocerebral complex of the insect brain, is best known for its pleiotropic functions in insect metamorphosis and reproduction [1]. Building on these basic functions, different classes of insects have apparently co-opted this hormone and its downstream signaling pathways and regulatory modules into functions that permit specific adaptations in their life cycles (e.g., diapause, [2]) or complex life histories (e.g., seasonal or caste polyphenisms, [3]). In the honey bee *Apis mellifera*, JH plays essential functions in caste determination during larval phases of development and controls the age-related transition from within-nest to foraging tasks in adult workers [4].

During larval development, the JH titer reaches a peak in the third to fourth larval instar and then drops to low levels at the beginning of the fifth instar in both castes. This peak in the early larval stages is particularly pronounced in queens [5], [6] and is an important factor for the caste-specific organ differentiation, especially the larval ovaries [7]. In adult bees, JH does not appear to play a role as a gonadotropin, as is the case in many other insects [8]. Rather, after a small peak of JH that initiates vitellogenin synthesis in the late pharate adult stage of queens [9], the JH titer stays at low levels throughout a queen's adult life cycle. In contrast, it has taken on a role in pleiotropically setting the physiological conditions for age-specific tasks in adult workers. In young workers, the hemolymph JH levels are low, and these bees exert intranidal tasks, primarily feeding the brood with secretions from their well-developed hypopharyngeal glands [10]. As the bees grow older and switch to performing more hazardous extranidal task, *viz.* foraging for nectar, pollen and water, their JH titers are typically increased [4].

Understanding regulatory mechanisms that underlie the fluctuations in the hemolymph JH titers is, thus, a major issue for coming to terms with honey bee sociality. Such regulation can occur via two ways, in the CA, via modulation of enzyme levels and enzyme activity in the biosynthetic steps of the sesquiterpenoid JH molecule, and via degradation and clearance of secreted JH in the hemolymph. JH-precursor manipulation and pharmacological inhibition experiments have shown that the final steps in JH synthesis are critically regulated in the honey bee CA [11], with their activity being modulated by biogenic amines [12] and also by the insulin-signaling pathway [13]. RNAi and partition assay experiments provided evidence that the honey bee JH esterase (AmJHE), but not the JH epoxide hydrolase (AmJHEH), is capable of degrading circulating JH [14], [15].

What is largely lacking in this picture is functional information on honey bee genes encoding enzymes of the JH biosynthetic pathway in the CA. To provide such information we searched for homologs of genes known to be involved in the JH biosynthetic pathway in *Drosophila melanogaster* and *Anopheles gambiae*. The expression levels of these genes were quantified by RT-qPCR in two contrasting situations of the honey bee life cycle. The first one comprised the fourth larval instar, where the JH titers in prospective queens are much higher than those in prospective workers [6]. The second contrasting situation concerned young adult workers performing intranidal tasks (nurses) versus older workers undergoing foraging tasks, i.e., two stages in the adult life cycle where JH titers are widely different [4], [16]. So as to then correlate the respective gene expression levels with the JH titers in these stages we also measured the general diameter of the CA and the diameters of CA nuclei as a proxy for CA activity.

## Results

### *In silico* analyses of JH biosynthetic pathway genes

Juvenile hormone biosynthesis involves the production of farnesyl pyrophosphate (farnesyl-PP) from acetyl-CoA via the mevalonic acid pathway, followed by converting farnesyl-PP into JH-precursors (farnesoic acid and methyl farnesoate) (Figure S1). To characterize candidate genes encoding enzymes in the JH biosynthetic pathway of honey bees, we used EST data generated from CA of *D. melanogaster* and *A. gambiae* [17] as queries in BLASTP searches to find orthologous sequences in the honey bee genome (version 4.0). EST libraries gave us information on the JH biosynthetic pathway genes that were actually expressed in these glands, as deduced by the detection of their respective transcripts. This useful information was important for the characterization of the expression of these genes in the CA-CC complexes of the honeybee.

Reciprocal matches corresponding to 25 enzyme-encoding sequences were found and the respective conserved functional motifs were identified. The predicted sequences identified in the honey bee genome (GBs), their corresponding orthologs in *A. gambiae* and *D. melanogaster*, as well as the predicted enzymes with their respective functions in the JH biosynthetic pathway are listed in Table 1. In accordance with prior *in silico* analyses [18] and in contrast to other insects [19], [20], we retrieved seven genes, putatively paralogs, that encode enzymes with a farnesyl diphosphate synthase (prenyltransferase) (FPPS) function. The predicted structure of these 25 genes is represented in Figure S2.

**Table 1:** Genes encoding enzymes of the JH biosynthetic pathway in *Apis mellifera*. Prediction based on BLASTP comparisons using *Anopheles gambiae* and *Drosophila melanogaster* sequences against the honey bee genome (version 4.0).

Enzyme	Symbol	Function	E.C. number	*A. gambiae ortholog*	*D. melanogaster ortholog*	*A. mellifera* ortholog	*E-value*
Methionine adenosyl transferase	MAT	Synthesis of S-adenosyl-L-methionine (AdoMet)	2.5.1.6	XM_307861	NM_164362	XM_623666	e-132
Adenosylhomocysteinase	AHC1	Hydrolysis of S-adenosyl-L-homocysteine (AdoHcy)	3.3.1.1	XM_311257	NM_078609	XM_391917	0.0
	AHC2					XM_624149	e-124
Adenosine kinase	AK	Phosphorylates adenosine	2.7.1.20	XM_307001	NM_168532	XM_391988	e-110
Acetoacetyl CoA thiolase	ACT	Condenses 2 molecules of Acetyl-CoA	2.3.1.9	XP_321828	NP_612094	XM_391843	2e-77
Hydroxymethylglutaryl-CoA redutase	HMGR	Reduces HMG-CoA to mevalonate	1.1.1.34	XP_307890	-	XM_623115	0.0
Hydroxymethylglutaryl-CoA synthase	HMGS	Condenses Acetoacetyl-CoA+acetyl-CoA	2.3.3.10	XP_315872	NP_725570	XM_397202	e-171
Mevalonate kinase	MK	Phosphorylates mevalonate	2.7.1.36	XM_319701	NM_176158	XM_624542	1e-30
Phosphomevalonate kinase	PMK	Phosphorylates phosphomevalonate	2.7.4.2	XP_310779	Q9VIT2	XM_001120920	1e-34
Isopentenyl-diphosphate delta-isomerase	IPPI	Isomerization of IPP to DMAPP	5.3.3.2	XP_321388	NP_650962	XM_001121223	4e-27
Farnesyl diphosphate synthase (prenyltransferase)	FPPS1	Sequential condensation of IPP with DMAPP and then GPP to form FPP	2.5.1.1/10	XP_308653	NP_477380	XM_001122575	2e-66
	FPPS2					XM_623583	8e-79
	FPPS3					XM_326224	8e-76
	FPPS4					XM_001122685	5e-59
	FPPS5					XM_624295	2e-59
	FPPS6					XM_001123325	1e-37
Citrate (*si*)-synthase	CS	Synthesis of citrate in the mitochondria	2.3.3.1	XM_320478	-	XM_393545	0.0
ATP citrate lyase	ATPCL	Synthesis of cytosolic acetyl-CoA from citrate	2.3.3.8	XM_319323	-	XM_623080	0.0
Mitochondrial citrate transport protein	MCTP1	Transports citrate from mitochondria to cytosol	-	XP_308964	NP_727450	NM_001010975	e-128
	MCTP2					XM_395934	e-110
Short-chain dehydrogenase	SCD	Oxidation of farnesol to farnesal?	-	XM_556135	NM_132467	XM_624031	5e-57
Crustacean “Farnesoic acid O-methyltransferase” homolog	FAMET	Function unclear	-	XP_318631	NP_611544	XM_623143	e-101
*Bombyx* JHA methyl transferase ortholog	MT	Transfers methyl group from AdoMet to farnesoic acid	-	XP_314173	NP_609793	XM_001119986	1e-40
Methyl farnesoate epoxidase (CYP15)	MFE	Oxidation of MF into JH III	-	XP_315675	NP_649151	XM_623572	5e-80

Enzyme Commission (E.C.) classification and enzyme functions are shown.

### Expression of JH biosynthetic pathway genes in different tissues of forager bees

**Figure 1:**
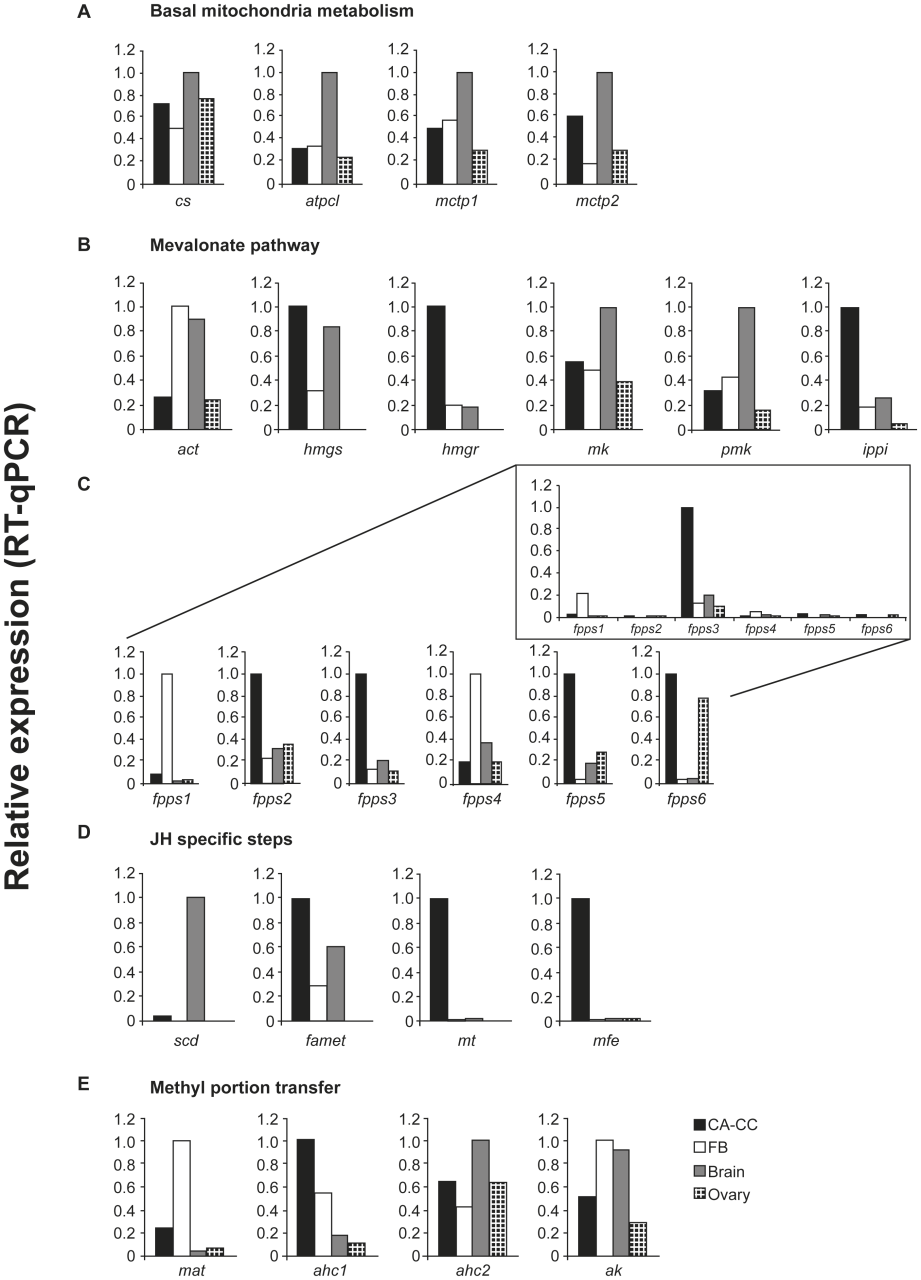
Expression of JH biosynthesis pathway genes in tissues from forager bees. Genes are grouped according to the function of the corresponding enzymes: (A) basal mitochondrial metabolism; (B, C) mevalonate pathway; (D) JH-specific steps; (E) methyl moiety transfer. Total RNA extracted from *corpora allata*-*corpora cardiaca* complexes (CA-CC), brain, fat body (FB), and ovaries were used for transcript quantification by real time RT-PCR (RT-qPCR). Each column in the graphs represents transcript levels in a single sample of 20–25 pooled CA-CC complexes, and 10 pooled brains, fat bodies and ovaries. The highest expression value for each gene was converted to 1. In the insert (C) the highest expression level among *fpps* genes was converted to 1.

The expression of the genes involved in JH biosynthesis (Table 1), except *fpps7*, was investigated in the CA-CC complex, brain, fat body and ovaries of forager bees (Figure 1). These genes are potentially involved in basal mitochondrial metabolism leading to Acetyl-CoA production (Figure 1A), and in the mevalonate pathway (Figure 1B, 1C), or specific steps of JH biosynthesis (Figure 1D, 1E).

Transcripts of all these genes encoding enzymes of the JH biosynthetic pathway were detected in the CA-CC complex (Figure 1). This is consistent with the high rates of JH biosynthesis in the CA and high titers of JH in the hemolymph of foragers [4]. The expression of several of these genes was undetectable, or detected at only basal levels in fat body (*fpps5*, *fpps6*, *scd*, *mt*, *mfe*,), ovaries (*hmgs*, *hmgr*, *ippi*, *fpps1*, *scd*, *famet*, *mt*, *mfe*, *mat*), or in the brain (*fpps1*, *fpps6*, *mt*, *mfe*, *mat*). Expression profiles of the FPPS codifying genes in tissues and organs of forager bees were compared separately. As *fpps3* turned out to be highly expressed in the CA-CC complex (Figure 1C - insert) this suggests that it is the *bona fide* farnesyl diphosphate synthase gene involved in JH biosynthesis in honey bees.

We then selected six genes, *mfe*, *mt*, *hmgr*, *ippi*, *fpp3* and *hmgs* for in-depth studies. As shown in Figure 1, these genes are highly expressed in CA-CC complexes and can, thus, serve as markers for comparing JH biosynthesis in honey bee castes and during development.

### JH biosynthesis gene expression in the CA-CC complex in relation to JH dynamics

**Figure 2:**
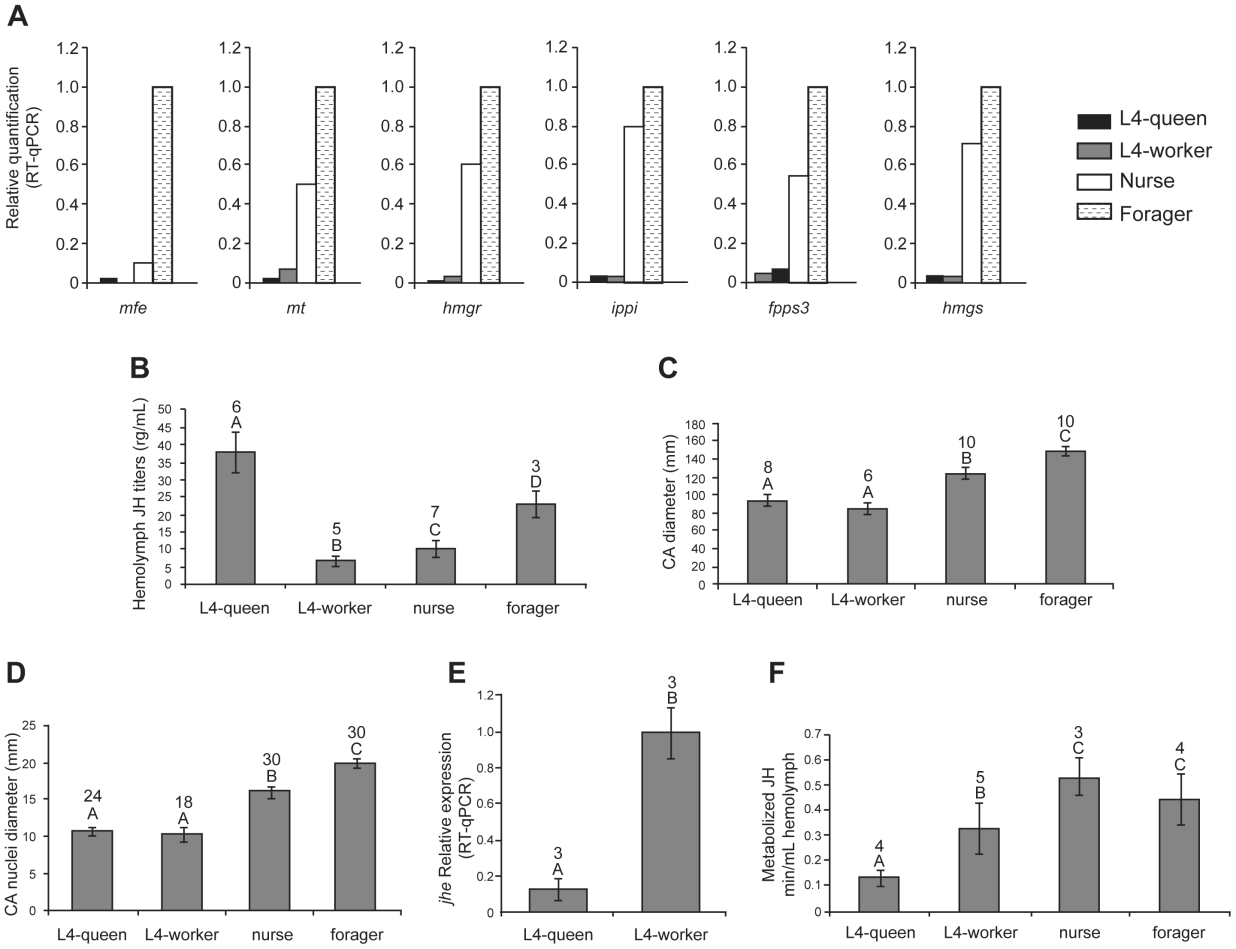
Transcript levels of JH biosynthesis pathway genes, JH titer, *corpora allata* (CA) size, and JH metabolism in honey bee larvae and adults. (A) relative expression levels of genes encoding enzymes of the JH biosynthesis pathway in CA-CC complexes; transcript levels were determined by RT-qPCR using a pool of 10 CA-CC pairs; *rp49* expression was used for normalization. The highest expression value for each gene was converted to 1. (B) Hemolymph JH titers measured by RIA. (C and D) CA diameter and diameter of CA nuclei measured by confocal microscopy following DAPI staining. (E) relative expression of the *jhe* gene, the major gene involved in JH metabolism, in the larval whole body. (F) JH metabolism (JHE activity) assayed by partition assay. Data refer to fourth instar (L4) queen and worker larvae, and two stages in the life cycle of adult workers (nurse and forager). Means ± SEM are represented in all panels, with sample sizes (N) shown above bars, except for panel 2A, where transcripts levels were quantified in single pools of CA-CC complexes. Different letters represent statistical differences. Statistical analyses: (2B, C and D) One-way ANOVA, *post-hoc* Holm-Sidak test, p≤0.001; (2F) One-way ANOVA, *post-hoc* Tukey test, p≤0.001; (2E) t-test: p≤0.001.

Transcript abundance of the six JH biosynthetic pathway genes, *mfe*, *mt*, *hmgr*, *ippi*, *fpp3* and *hmgs* was contrasted to CA size and the hemolymph JH titers and metabolism in adult workers performing intranidal versus forager tasks, and also in fourth instar queen versus worker larvae (Figure 2).

In the case of adult workers, the RT-qPCR analysis of a sample consisting of 10 CA-CC complexes suggested that transcript abundance for all these genes was higher in foragers than in nurses (Figure 2A). Although this statement cannot be supported statistically because it is based on a single-sample analysis, we found that these differences correlated well with the high hemolymph JH titer in foragers (Figure 2B).

Furthermore, when comparing CA size (Figure 2C) and the size of CA nuclei (Figure 2D) these were also significantly larger in foragers. Therefore, the expression of these six genes in adult workers was correlated with JH hemolymph titer and CA size.

In contrast to our findings for adult workers, there was no such correlation in the fourth instar larvae. In fact, while the JH titer in queen larvae was considerably higher than in worker larvae (Figure 2B), in accordance with literature data [6], there were no apparent differences in transcript abundances for any of the six JH biosynthetic pathway genes (Figure 2A). Quite strikingly, transcript abundances for all these genes seemed rather low when compared to those seen in CA-CC complexes of adult bees (Figure 2A). Furthermore, we also did not observe any significant difference in general CA size (Figure 2C) and CA nuclei diameters in these fourth instar queen and worker larvae (Figure 2D), this suggesting that JH titer regulation in fourth instar queen and worker larvae does not depend on the differential expression of JH biosynthesis genes and is also independent of CA size. In more general terms, this would mean that the gene-regulatory network underlying JH synthesis in the CA-CC complex and the consequent modulation of the JH titer follow different rules in larval versus adult honey bees. To follow up on this question we next looked at JH degradation as a means of creating divergence in JH hemolymph titers.

### JH esterase gene (*jhe*) expression and JH degradation activity in hemolymph

In a previous study [14] we had shown that the *jhe* gene, which is predicted to encode a putative JH esterase, has an expression pattern in the fat body that runs exactly opposite to the JH titer. We therefore investigated *jhe* transcript abundance in queen and worker larvae (Figure 2E) and contrasted these with the esterase-related JH degradation activity, measured by a JH partitioning assay, in the respective hemolymph samples (Figure 2F). In accordance with our predictions, *jhe* transcript abundance was considerably lower (p≤0.001) in fourth instar queen larvae when compared to same instar workers (Figure 2E), and JH degradation activity was also significantly lower in queen hemolymph (Figure 2F), this indicating that JH degradation is an important regulatory factor for the physiological hormone titer. It is also worthy of note that there was no significant difference in JH esterase activity in the hemolymph samples from adult workers (Figure 2F), lending further support to our hypothesis concerning differences in mechanisms underlying JH biosynthesis and titer regulation in larvae versus adult honey bees.

### Genes encoding JH biosynthesis enzymes respond to differential feeding during adulthood

**Figure 3:**
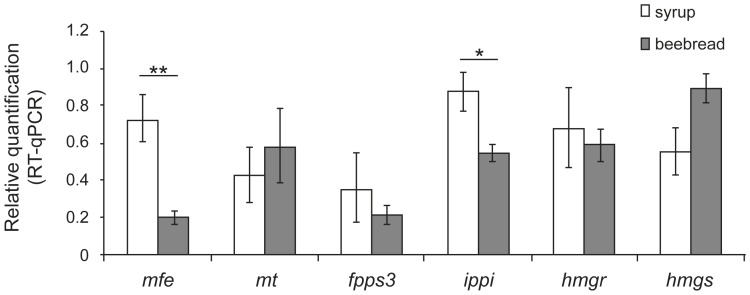
Effect of diet on transcript levels of genes encoding enzymes of the JH biosynthesis pathway and expressed in CA-CC complexes. Newly-emerged workers were kept for 7 days in cages and fed on a protein-enriched diet (beebread plus syrup), or on a pure carbohydrate diet (syrup only). Each bar represents the mean ± SEM of three independent samples, each consisting of eight CA-CC pairs. (**) t-test; p≤0.001; (*) t-test; p≤0.05.

Hive bees mainly feed on a protein-rich diet, processed from the pollen stores in the hive, whereas foragers prefer an almost pure carbohydrate diet [21], [22]. As nutrition is linked to behavior and aging, we investigated whether and how diet affects the transcription levels of JH biosynthesis pathway genes. Newly emerged workers were kept for seven days in wooden cages and fed with a beebread/syrup diet (beebread) or with syrup only. For both groups we then assessed the transcript levels of six candidate genes (*mfe*, *mt*, *fpps3*, *ippi*, *hmgr* and *hmgs*) in the CA-CC complexes (Figure 3). We found that only *ippi* and *mfe* were differentially expressed under the two dietary conditions, both being more expressed in the syrup fed group (Figure 3). This is in agreement with the above reported findings on the transcript levels of the *mfe* and *ippi* genes (Figure 2A), suggesting that these genes are upregulated in foragers that feed on a carbohydrate-rich diet and typically have a high JH titer, when compared to nurse bees that feed on a protein-rich diet and have a low JH titer.

### RNAi-mediated knockdown of methyl farnesoate epoxidase (*mfe*) gene function

The *mfe* gene, which putatively encodes an enzyme in the final JH biosynthesis steps, was chosen for a functional gene disruption assay. Three groups of newly emerged workers were set up in this experiment, one was injected with target *mfe* dsRNA, the second one received GFP dsRNA as an off-target dsRNA control, and the third group was left untreated (control). Seven days after the treatment the bees were collected, total RNA was individually extracted and used to check knockdown efficiency.

**Figure 4:**
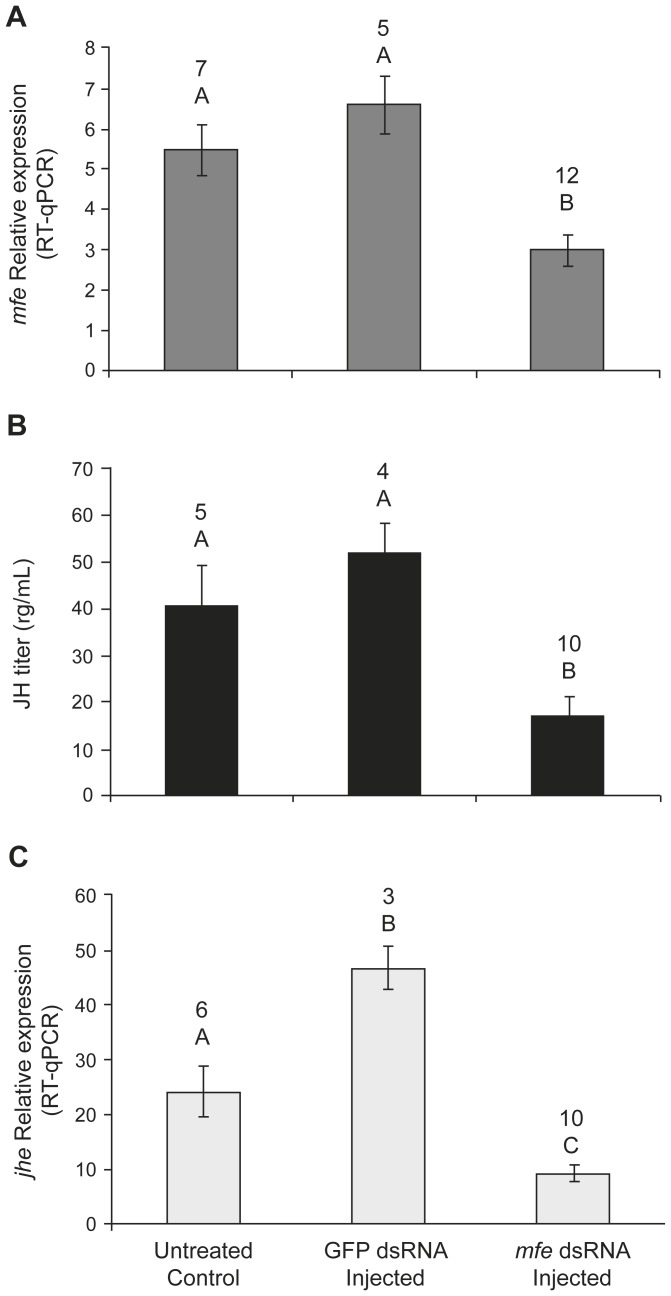
Effects of RNAi-mediated silencing of methyl farnesoate epoxidase (*mfe*) gene function on major control genes of JH biosynthesis and metabolism and on the hemolymph JH titer. Newly-emerged adult workers were injected with 3 µg *mfe* dsRNA or GFP dsRNA or left untreated. The bees were collected 7 days after the treatment. (A) *mfe* transcript levels determined by RT-qPCR using whole body RNA. (B) JH hemolymph titers measured by RIA. (C) *jhe* transcript levels determined by RT-qPCR using whole body RNA. *rp49* was used for normalization. Each bar represents the mean ± SEM of independent samples; sample size is shown above bars. Different letters represent statistical differences assessed by One-way ANOVA followed by Tukey's *post hoc* test; (p≤0.001 in A and C; p = 0.003 in B).

The *mfe* dsRNA-injected group showed an up to 50% reduction in *mfe* mRNA abundance when compared to the two other groups (Figure 4A). As this gene is primarily expressed in the CA-CC complexes (Figure 1D), this is a good indication of the silencing efficiency in the targeted organ. A similar level of reduction was also seen in the hemolymph JH titer (Figure 4B), thus reinforcing the prior evidence and directly linking this gene to the regulation of the JH titer in adult honey bees. Further support also came from the RT-qPCR assays run on *jhe* gene expression, which was significantly decreased in the *mfe*-knockdown group (Figure 4C). But here the link between the two genes probably goes through the regulation of the hemolymph JH titer, as in a previous study [14] it has been shown that a topical application of synthetic JH-III can induce *jhe* transcription, and that *jhe* knockdown bees had higher JH titers in their hemolymph. This RNAi assay on *mfe*, an apparently critical gene in the JH biosynthetic pathway, thus lends further support to the hypothesis that, in adult honey bees, the regulation of the JH titer associated with division of labor in workers involves the transcriptional regulation of JH biosynthesis-specific genes, this contrasting with the situation in the larval stages, where the regulation of a gene encoding a JH-degrading esterase (*jhe*) would be more crucial for the divergence in queen/worker phenotype development.

## Discussion

Two hallmarks of the social organization of honey bees, caste development and age-related division of labor of adult workers, are controlled by the hemolymph JH titer. Whereas the dependence of these processes on JH received much attention and is worked out in descriptive and mathematical models [23], [24], the mechanisms underlying JH titer regulation are not yet completely known. The steady-state hemolymph JH titer is modulated by the activity of enzymes in the biosynthetic pathway in the CA (Figure S1), or by enzymes involved in its degradation which in honey bees occurs primarily via a JH esterase, or by a combination of both. Whilst the genes encoding JH degrading enzymes and their functionalities have been fairly well characterized in honey bees [14], [15], this is not so for the JH biosynthesis steps.

### Annotation and expression of JH biosynthesis genes

Based on computational analysis of *A. mellifera* candidate genes in JH biosynthetic pathway [18] and using a database comprising ESTs from insect CA transcriptomes [17], [20], [25]–[27] 25 genes were found and annotated in the honey bee genome. Amongst these, the farnesyl diphosphate synthase (*fpps*) deserves special attention due to the high number of paralogs. While the *fpps* gene is a single-copy gene in all sequenced Diptera [17], it is represented by seven copies in the honey bee genome. The *fpps* genes are not a unique case of gene duplication or gene family expansion in the honey bee genome, as this also happened in another 60 genes, and these have been interpreted as possible signatures of solitary to social life transitions in bees [18]. Our RT-qPCR results now showed that all these *fpps* paralogs are expressed in the retrocerebral CA-CC complex, and *fpps3* seemed to be the most expressed one (Figure 1C - insert), thus suggesting that it is a *bona fide* candidate for an FPPS function in the JH biosynthetic pathway.

The expression of 24 of the 25 annotated JH biosynthesis genes was assayed in RNA extracts from CA-CC complexes, remaining brain tissue, fat body, and ovary. All genes were expressed in the CA-CC complexes, placing in evidence their putative role in JH biosynthesis. Transcription for most of the genes was also evidenced in the other tissues, which is not surprising, considering that the mevalonate pathway enzymes are required not only for JH production but also for the production of other bioactive terpenoids and for the farnesylation of proteins [28]. In contrast, two genes involved in JH-specific steps, those encoding *O*-methyltransferase (*mt*) and methyl farnesoate epoxidase (*mfe*), were specifically expressed in the CA-CC complexes.

Methyl farnesoate is the immediate precursor of JH in insects [29] and has hormonal functions in crustaceans [30]. Farnesoic acid *O*-methyltransferase (FAMeT) and JH acid *O*-methyltransferase (JHAMT) (called here MT) are described as catalyzing the methylation of farnesoic acid to methyl farnesoate with S-adenosyl-L-methionine functioning as cofactor [25]. In the fruit fly, *D. melanogaster*, a recombinant FAMeT (rFAMeT) was cloned, expressed, and a specific antiserum was generated. Immunohistochemical analysis confirmed the presence of FAMeT in the CA portion of the ring gland, but rFAMeT presented no enzymatic activity in a radiochemical assay, inferring that *D. melanogaster* FAMeT has little, if any role in sesquiterpenoid biosynthesis [31]. Notwithstanding, the Dm*JHAMT* gene is predominantly expressed in the fly CA. Moreover, the recombinant protein was capable of catalyzing the methylation/esterification of JH-III acid or farnesoic acid in the presence of S-adenosyl-L-methionine [32]. Subsequently, Marchal et al. [33], using the locust *Schistocerca gregaria* as a model, showed that JHAMT, and not FAMeT, is the enzyme involved in JH synthesis. In the honey bee genome we found orthologs for both, *famet* and *mt* genes, and when assaying their expression by means of RT-qPCR we found that *mt* transcripts were specifically detected in CA-CC complex, whereas *famet* was transcribed more ubiquitously, as is also the case in the highly eusocial stingless bee *Melipona quadrifasciata* [34]. This leads us to infer that *mt* and not *famet* is the gene encoding a functional methyltransferase activity in the honey bee CA.

The *mt* gene (*AmJHAMT* gene) was recently assayed in terms of expression patterns and functionality in honey bee larvae and pupae [35]. It was observed that in these whole body RNA extracts the *AmJHAMT* gene expression profile during the fourth and fifth larval instars does not follow the modulation in JH levels. For instance, low *mt* transcript levels coincided with high JH levels in the fourth larval instar, and this was inverted in the fifth instar. Furthermore, as revealed by our data, *mt* transcript abundance in fourth instar queens and workers stood in contrast with the respective JH levels. Therefore, neither of these studies could find a positive correlation between levels of *AmJHAMT* transcripts and JH in the larval stages. Our hypothesis is that the larval JH titer is primarily regulated by JHE activity, and this is supported by evidence showing an increase in *jhe* transcripts in fourth and fifty instar worker larvae [14].

### Methyl farnesoate epoxidase (MFE) versus JH esterase (JHE) – different modes of JH titer control in caste development and age-related division of labor?

The steady-state JH titer in hemolymph is a snapshot picture resulting from a balance in JH synthesis and release from the CA, and degradation by metabolizing enzymes. The regulation of synthesis has been pointed out as an important step in this process. So as to understand these processes at the level of transcriptional control and functionality of certain key genes in honey bees we looked at two contrasting situations in the honey bee life cycle, i.e., caste development and age-related division of labor.

For caste development we put our focus on the fourth larval instar, which is the stage when the JH titer in queen and worker larvae shows maximal differences [5], [6]. Earlier results obtained by a radiochemical assay of JH biosynthesis *in vitro* showed that MFE activity is not only the final step of JH synthesis in honey bee CA, but is also a limiting factor for JH production in CA of honey bee larvae [36]. The honey bee MFE is clearly the ortholog of a P450 enzyme (CYP15A1), which catalyzes the production of JH from methyl farnesoate in *D. punctata* [26]. Nonetheless, *mfe* transcript abundance is basal, or undetectable in the CA of fourth instar queen and worker larvae (Figure 2A), this raising the question of how the hemolymph JH titer could be so much higher in queen larvae (Figure 2B). One possibility would be CA size, but these turned out to be similar for queen and worker larvae (Figure 2C, 2D), and in this respect, honey bee larvae are similar to *Manduca sexta*, where CA volume does not correlate with the hemolymph JH level [37]. In late fifth instar *A. mellifera* larvae, CA volume varies very little [36], and this is also the case for *M. sexta*, where CA volumes were found to be much more related to larval age/size than to JH synthesis activity [38].

With larval CA size out of the question, the factor left was JH degradation and, indeed, this was found to be the case, as revealed by a much lower rate of JH metabolism in hemolymph of queen larvae (Figure 2F). These low rates were furthermore in accordance with reduced expression levels of the *jhe* gene (Figure 2E). For JH titer regulation in the fourth larval instar, the transcriptional control of *jhe*, a gene driving JH degradation in honey bees [14] should, thus be the primary critical factor. This assumption is in accordance with the respective rates of JH biosynthesis measured in a radiochemical *in vitro* assay [39], showing that JH biosynthesis rates in this instar are still very similar in queen and worker larvae, whereas their JH hemolymph titers are maximally different [6]. In the fifth larval instar, this regulatory mode may, however, change, as JH biosynthesis rates drastically increase in queen larvae until the end of the feeding phase, but concomitantly, the JH titer in hemolymph drops to basal levels, thus indicating increased clearance of JH also in queen larvae.

In adult workers, the results of the JH titer measurements were consistent with previous results showing that foragers have higher hormone levels than the younger nurse bees [4], [16], [40], [41]. As we could show, these differences in steady-state JH levels were not due to differences in JH degradation, but seemed associated with the expression levels for all the six JH biosynthesis pathway genes studied herein (Figure 2). This suggests that all these genes are upregulated once workers switch from performing within-hive tasks to become foragers. Interestingly, the gene that showed a high relative expression was *mfe*, and it was also this gene that was significantly upregulated in caged workers fed on a carbohydrate-rich diet (Figure 3). The expression of this gene seems, thus, not only linked with the age-related behavioral switch to foraging, but also reflects the nutritional physiology of foragers [22], even when these bees are kept in cages and cannot fly. Among the other genes assayed with respect to the bees' nutritional conditions, only *ippi* was also significantly upregulated in syrup-fed bees. But whereas there is strong experimental evidence for MFE as a key factor in controlling the JH titer [36], this is not the case for *ippi*, which encodes an isopentenyl diphosphate isomerase in an alternative route of the mevalonate pathway (see Figure S1).

Bees fed on a pure carbohydrate diet did not accumulate vitellogenin in their hemolymph [42], and, rather, it is the depletion of vitellogenin which has been linked to the hive bee-to-forager transition and predicted in the double repressor hypothesis [43] as an activation of an allato-regulatory central nervous system pathway. This hypothesis has since been validated by manipulation of JH levels, as well as by knocking down vitellogenin transcript levels by dsRNA injection [44]–[46], all these experiments confirming that the increase in JH titer at the transition from hive bee to forager behavior is contingent on a reduction in vitellogenin levels. Herein we now showed that the expression of *mfe*, a key gene in JH biosynthesis, is also directly contingent on the nutritional state, thus adding an extra component to this double repressor circuitry.

As demonstrated by several authors [47], [48] the process of behavioral maturation in bees is complex and influenced by different intrinsic and extrinsic factors, with age and nutritional state being of prime importance. The interference of the nutritional status with the onset of foraging is a well-described fact [49], [50], and seemingly, this involves the insulin-signaling pathway. Ament et al. [51] showed that an insulin receptor encoding gene, *InR isoform 1*, is upregulated in the abdomen of honey bee workers in response to a sugar diet, thus connecting the insulin-signaling pathway with behavioral maturation.

So as to better understand the connection between *mfe* expression and JH titer, we performed an experiment silencing *mfe* gene function by dsRNA injection. This experiment showed that not only was the JH titer level reduced in *mfe* knockdown bees, as expected, but that this also led to a reduction in *jhe* transcript abundance, indicating that JH degradation would also be reduced. A decrease in *jhe* transcript levels was detected as early as five days after the injection, but was more consistent on day 7 (Figure 4). We hypothesize that this may represent part of a feedback circuitry, whereby circulating JH may directly control its main degrading enzyme [14], so as to prevent excessive variation in steady-state hormone levels, this further illustrating the complexity in transcriptional control within the JH biosynthetic/degradation pathways in relation to age-related division of labor in honey bees.

MFE activity is evidenced as a limiting factor for JH production in the CA of honey bee larvae [36], but regulation of JH biosynthesis may occur to some extent at each one of the sequential steps in the biosynthetic pathway. Interference in this pathway, as well as in JH degradation via JHE activity [14] may affect the temporal fluctuation of JH titer. But these may not be the only factors. Recent *in silico* analyses performed in our laboratory revealed target binding sites for several known microRNAs in genes encoding enzymes of the JH-biosynthetic pathway. Importantly, target binding sites for twelve of these microRNAs (among them ame-miR-375-3p, ame-miR-3794-5p and ame-miR-6059-3p) are shared by the *mfe*, *jhe* and *jheh* genes, suggesting that these may be co-regulated. We also observed that each of these genes may be targeted by exclusive microRNAs. This emerging array of noncoding small RNAs, which regulate gene expression at the post-transcriptional level, adds a novel interesting twist to the mechanism underlying JH titer regulation and clearly deserves further attention.

## Conclusion

We annotated 25 genes of the JH biosynthetic pathway in the honey bee genome and assayed the expression levels of 24, showing that several of these are prominently expressed in CA-CC complexes, when compared to other tissues. Six genes comprising representatives of the mevalonate pathway, as well as of the JH-specific steps were selected for transcript abundance measurements in two contrasting situations of the honey bee life cycle, the fourth larval instar with its caste-specific differences in the JH titer, and in adult workers, which show task-related JH titer differences. This revealed that the transcriptional regulation of a methyl farnesoate epoxidase-encoding gene (*mfe*) involved in JH biosynthesis in the CA, and of a JH esterase encoding gene (*jhe*) involved in JH degradation, should be key events for the control of JH levels in the hemolymph. Interestingly, these genes appear to be regulated in a different fashion in the fourth larval instar, when compared to adult workers. Whereas in the fourth larval instar the JH titer was closely correlated with *jhe* transcript levels, in the adults this was the case for *mfe*. For adult workers we could furthermore show that *mfe* expression is also contingent on the nutritional status. This, together with the finding that *jhe* transcript levels were reduced in workers that had their *mfe* gene function diminished by RNAi, makes a case for the caste- and stage-specific complexity in the regulation of the hemolymph JH titer in honey bees. Probably this is what is expected of a pleiotropic hormone, and this could also be the center court for the evolvability of its functions in the diversity and plasticity of insect life cycles.

The molecular pathway differences underlying JH titer regulation in honey bee caste development versus age-related division of labor may reflect differences in selection pressures on key enzymes during the evolution of sociality. This may not only be reflected in the expansion of the *fpps* genes in the honey bee, as well in synonymous versus non-synonymous changes in the sequences of other genes involved in JH biosynthesis and metabolism. Both aspects could now be investigated by comparing these genes across bee genomes currently under study. In particular studies intending to investigate the co-option and changing roles of JH in social insects under a phylogenetic hypothesis should consider measuring the transcript levels of *mfe* and *jhe* orthologs in addition to the respective JH hemolymph titers.

## Materials and Methods

### Bees

Workers and queens of Africanized hybrids of the honey bee, *A. mellifera*, were collected from hives maintained at the Experimental Apiary of the University of São Paulo at Ribeirão Preto, Brazil. Queens were obtained by a standard apicultural queen rearing technique, whereby first instar female larvae were transferred to artificial queen cells containing a drop of royal jelly and then introduced into a queenless colony to be reared until the desired stage [9]. Newly emerged workers were paint-marked, introduced into queenright hives and subsequently collected according to the activity they were performing: nourishing larvae (nurse bees, between 7 and 10 days old), or foraging for pollen (older workers entering the hive carrying pollen).

### DAPI staining of CA-CC complexes

To assess size variation in the CA-CC complex as a possible indicator of JH synthesis levels, the CA dissected from fourth instar queen and worker larvae and from adult workers (nurses and foragers) were stained with DAPI [37]. Briefly, CA-CC complexes were dissected in PBS (137 mM NaCl, 8.1 mM Na_2_HPO_4_, 1.47 mM KH_2_PO_4_, 2.68 mM KCl), fixed in 4% paraformaldehyde in PBS for 30 min at room temperature and washed several times in PBS. The complexes were incubated in DAPI dissolved in PBS (1∶4000 w/v) for 4 min at room temperature, washed several times in PBS and mounted in 80% glycerol in PBS for analysis by confocal microscopy (Leica TCS-SP5 Laser Microscope; Leica Microsystems, Wetzlar, Germany). Measurements were taken on CA from ten foragers, ten nurses, eight fourth instar queen-destined larvae and six fourth instar worker-destined larvae. In total we measured the diameters of 34 CA and of 102 CA nuclei. The data were analyzed by one-way ANOVA followed by a *post-hoc* Holm-Sidak test at a significance level of p≤0.05.

### JH synthesis candidate genes - annotation and primer design

The honey bee genome database (http://www.hgsc.bcm.tmc.edu/projects/honeybee/) (version 4.0) downloaded to our local server (http://zulu.fmrp.usp.br/beelab) was searched for sequences encoding enzymes of the JH biosynthetic pathway, using *Drosophila melanogaster* and *Anopheles gambiae* orthologs as queries. The predicted protein sequences obtained by BLASTN analyses were confirmed by investigating the presence of specific motifs. Primer3 software [52] was used to design specific primers (Table 2) for quantitative PCR analysis. The structural architecture, including intron/exon boundaries of these genes, was manually annotated against the honey bee genome sequence using Artemis 7.0 software [53] implemented in a LINUX server.

**Table 2:** Primers for assaying JH synthesis pathway genes, and for a JH metabolism gene (*jhe*). *rp49* was used as reference gene.

Gene	Primer forward (Sequence 5′-3′)	Primer reverse (Sequence 5′-3′)	Amplicon length (nt)
*rp49*	CGTCATATGTTGCCAACTGGT	TTGAGCACGTTCAACAATGG	150
*jhe*	GTTATCGCTTCTGATATGGCT	GATGGGAAATAGGTACCGAC	121
*act*	GGCCTCAAACGACTCTTCAA	CGTGCCAGTGGTGTAAGTTG	163
*hmgs*	CCGTATCTTGGGTAGAAAGC	GATGATCTCACTCCACGATC	172
*hmgr*	CCTGCACAGAATGTTGGAAG	GTGCAGGAAGAATAGTTCCG	178
*mk*	TTCCGCTTCTTTTGCAGTTT	TTCCAGATGGATTACCATGC	155
*pmk*	CTCATTGGGCCAAATCTCAT	TATCTAGAGCCGCACGACAA	147
*ippi*	GCTGAAGCTACTTTGCAGCTA	GCTCGATGAAGTAAAACTTGTCC	137
*fpps1*	GAAGATCAGTACCAATTGTAATC	GTTGGTTGATTTCGTCGCATG	183
*fpps2*	GAGATCAGTATCGCTTATAGTC	GTTGGTTGATTTCGTCGCATG	174
*fpps3*	GGAAGACGAAGATCATATCACT	GTTGGTTGATTTCGTCGCATG	175
*fpps4*	GCGCATGGTGAATGTTTAGA	CGAAACGCATAGCAAGACTG	186
*fpps5*	CGAAAGGAGGAAAACGAAGA	CTGCCATTGTGTGAACTGCT	140
*fpps6*	CATCGATAGATTCGGGAAAGA	ATCGATTATGCCAGCGAAAC	185
*scd*	AGAGCTATCGCTGCTCTTGC	TGTGGGAAATGATGCTAACG	150
*famet*	GTAAAGAAGGTGAGCCAAGTG	CGATTAACCATTCACCAGAAG	111
*mt*	CCTTCACTGGTGCCAAAACT	TGGCCTATATCGAGGATTCG	140
*mfe*	GGAATCATTTCTTGCGGAGA	GTTATGCGCGCTATGGAAAT	145
*cs*	AATGAATGGTTTGGCTGGAC	GAACGACTTGACCGCTCTTC	152
*mctp1*	TTGATTGTTTTGTGCGCATT	TGTCAACACCACCAAGGAAA	150
*mctp2*	GAAATTTGGCTTCTGGTGGA	CAATCACCCAGACCTTTGAA	133
*atpcl*	CTGGCGCTTATGTTCCTGAT	ACCAAGTTCCCTAGCCCAAT	152
*mat*	TGCAAAAGTGGCTTGTGAGA	GCCAATCGAATCCTTTTGAA	152
*ahc1*	TGGATGAAGCATCACGAAAA	TGCAACATCAATTTCGCAGT	143
*ahc2*	GCCGAAGATAAGCCATTGAA	CAGCAGCCACTTCATTTTGA	154
*ak*	GCAAATGATGCAATTCTTGCT	ATTTGGTTTCCCCAAGAACC	141

### Tissue dissection, RNA extraction and cDNA synthesis

For comparative analysis of the transcript levels of genes encoding enzymes putatively involved in the JH biosynthetic pathway, foragers were dissected in ice-cold Ringer solution to retrieve the CA-CC complex. Due to the difficulty of quickly dissecting individual CA, each gland pair was removed together with the adhering CC. The CA-CC total RNA from foragers was extract using the GenElute™ Mammalian Total RNA Kit (Sigma). After dissecting remnant brain tissue, fat body, and ovaries, these tissue samples were transferred to 1 ml of Trizol Reagent (Invitrogen) and stored at −80°C until RNA extraction following the manufacturer's instructions. CA-CC complexes from fourth instar queen and worker larvae were also dissected in Ringer solution and RNA was extracted by homogenization in Trizol reagent.

RNA quantity and quality was assessed spectrophotometrically using a Nanodrop-1000 system (Thermo Scientific, Wilmington, MA, USA). Aliquots of 1 µg total RNA were treated with DNase I (Promega) before first-strand cDNA synthesis by reverse transcription using SuperScript II Reverse Transcriptase (Invitrogen) and an oligo(dT)_12–18_ primer (Invitrogen) following the manufacturer's instructions.

### Quantitative Real-Time RT-PCR (RT-qPCR)

By means of RT-qPCR assays we quantified the expression of a set of 24 candidate genes encoding enzymes putatively involved in the JH biosynthetic pathway. The assays were run in a 7500 Real Time PCR System (Applied Biosystems, Wilmington, MA, USA) under the following conditions: 50°C for 2 min, 95°C for 10 min and 40 cycles at 95°C for 15 s and 60°C for 1 min. The quantification was carried out in 20 µl reaction volumes containing 10 pmol of each primer, 10 µl SYBR Green Master Mix 2X (Applied Biosystems), 1 µl of cDNA, and water to complete the volume. The specificity of the primers was checked by sequencing of the amplicons. Primer efficiency (E) was calculated based on the slope of a standard curve (E = 10^(−1/slope)^) generated from serial 1∶10 dilutions of cDNA. The *rp49 ribosomal protein* gene (GenBank accession number AF441189), also known as *rpl32* (GenBank accession number NM 001011587.1), was used as reference gene based on prior validation experiments [54]. Data were analyzed according to the comparative threshold cycle (Ct) method, in which the amount of the target transcript is normalized to the reference gene and relative to a calibrator and is given as 2^−ΔΔCt^ fold change.

### JH titer quantification by radioimmunoassay (RIA)

Hemolymph was collected from fourth instar larvae (queens and workers) and from adult workers (nurses and foragers) using a microcapillary, and 1 µl aliquots were immediately transferred to 0.5 ml of acetonitrile in a Teflon-lined screw cap vial and stored at −20°C until analysis. The JH extraction from hemolymph followed the liquid-phase separation protocol established by Huang et al. [55], and the antibody used was developed by Goodman et al. [56]. The protocol for the use with honey bee samples is described in full detail in Hartfelder et al. [57]. Based on standard curve values, JH titers were calculated by non-linear four-parameter regression (ImmunoAssay Calculations spreadsheet, Bachem, Bubendorf, Switzerland) and expressed as JH-III equivalents (pg/µl hemolymph). The data were analyzed by one-way ANOVA followed by a *post-hoc* Tukey test, at a significance level of p≤0.05.

### Partition assay for JH esterase (JHE) activity

JHE activity was estimated by a partition assay developed by Share and Roe [58] which uses ^3^H-labeled JH as substrate. The potent and specific JH esterase inhibitor 3-octylthio-1,1,1-trifluoro-2-propanone (OTFP) was used to measure JH degrading activity in the honey bee hemolymph. Hemolymph samples were collected with a microcapillary from an incision in the integument of fourth instar larvae (queens and workers) and adult workers (nurses and foragers), and centrifuged at 5,000× *g* for 5 min at 4°C. Hemolymph (10 µl) was mixed with 10 µl PBS and incubated with 1 µl ethanol or 1 µl 2 mM OTFP diluted in ethanol for 10 min at 30°C, followed by incubation at 30°C for 45 min with 100 µl of 6.08 nmol radiolabeled JH-III ([10–^3^H(N)]-JH-III, specific activity 11.8 Ci/mmol; PerkinElmer Life Sciences) diluted in PBS, to give approximately 6200 cpm per assay. To stop JHE activity at the end of the assay, 50 ml of methanol, water and ammonia solution (10∶9∶1, v/v) were added and the mixture briefly stirred before adding 250 µl isooctane to partition non-hydrolyzed JH-III into the organic phase. A 75 µl aliquot of the aqueous phase containing the water-soluble JH degradation product was taken for liquid scintillation counting (LS 6500 Scintillation Counter Beckman, Ramsey, MN, USA) in Optiphase HiSafe3 cocktail (Packard). Each sample was assayed in triplicate. JHE activity was expressed as nmol JH acid produced per min per ml hemolymph.

### Differential feeding experiment of adult workers

This experiment was done to investigate the effects of food intake on the transcript levels of JH biosynthesis genes. Newly-emerged workers (n = 20) were kept for seven days in 8×11×13 cm screened wooden cages at 34°C and 80% humidity, where they received either syrup (50% sugar in water), or beebread (pollen collected from cell combs) plus syrup (30% beebread in syrup) diets. In both treatments, the bees received water and the respective diet *ad libitum*. Three pools of eight CA-CC complexes were dissected from bees submitted to each treatment and used for total RNA extraction with GenElute™ Mammalian Total RNA Kit (Sigma); cDNA synthesis was done as described above.

### RNA interference for silencing *mfe* gene in adult workers

The function of the methyl farnesoate epoxidase-encoding gene, *mfe* (Official Gene Set 3.2, GB15634), in the JH biosynthetic pathway was investigated by an RNAi experiment performed in adult workers based on a protocol established by Amdam et al. [59]. Briefly, dsRNA was synthesized using a RiboMax Large Scale RNA Production System – T7 (Promega), following the manufacturer's protocol and using gene-specific primers (dsMFE-f: TAATACGACTCACTATAGGGCGAGATCAATGTGGTCTCAGTG and dsMFE-r: TAATACGACTCACTATAGGGCGATCGTGATCTACCTACTACG). The dsRNA was purified using Trizol Reagent and maintained at −80°C until use.

For injection, cold anesthetized newly-emerged workers were immobilized on a piece of styrofoam where they were kept at 8°C during the procedure. Two groups of these bees were injected with double-stranded mfe RNA (*mfe* dsRNA) or with a dsRNA for Green Fluorescent Protein (control) (GFP dsRNA). One µl of the dsRNA solution (3 µg dsRNA per µl Ringer saline) was injected laterally into the abdomen between the 5th and 6th tergites. A third group served as untreated control. All groups were kept in 8×11×13 cm screened wooden cages at 34°C and 80% humidity where they were fed with syrup and water *ad libitum*. After 7 days, 2 µl of hemolymph were collected from an incision in the thorax of cold anesthetized bees, to be used for JH titer analysis by RIA. The remaining whole body was stored in Trizol Reagent (Invitrogen) and maintained at −80°C until use. After RNA extraction, 3 µg of DNase I-treated total RNA was used for cDNA synthesis. A total of 12 bees injected with *mfe* dsRNA, 5 GFP dsRNA injected bees and 7 untreated bees were analyzed for *mfe* expression by means of RT-qPCR assays.

After validating the knockdown, hemolymph samples of those bees showing a significant decrease in *mfe* transcript levels (one-way ANOVA post-hoc Tukey test and significance at p≤0.05) were used for measuring JH titers.

## Supporting Information

Figure S1 **JH-III biosynthetic pathway (modified from Bellés et al. 2005 Annu Rev Entomol 50: 181–99, and Noriega et al. 2006 Insect Biochem Mol Biol 36:11 366–374). The underlined enzymes correspond to the genes whose expression is shown in** **Figure 1B–E** **.**
(TIF)

Figure S2 **(A) Representation of the** ***A. mellifera*** **genes encoding enzymes of the JH biosynthetic pathway.** Exons and introns are represented by non-scaled boxes and lines, respectively. Nucleotide numbers are indicated to provide a general estimation of gene size. The direction of transcription is indicated by an arrow. (B) Architecture of the genes encoding Farnesyl diphosphate synthase (FPPS). Exons are named by their respective lengths. Lines between exons indicate intron and their respective length is indicated. The direction of transcription is indicated by a black arrow. The dashed arrows indicate that part of the sequence is not available in the honey bee genome database (version 4). Arrowheads indicate primers position.
(EPS)
