# Supplementary figures and images for: Juvenile Hormone Biosynthesis Gene Expression in the corpora allata of Honey Bee (Apis mellifera L.) Female Castes
Source: PLoS One. 2014 Jan 29;9(1):e86923. doi: 10.1371/journal.pone.0086923 (PMC3906101; doi:10.1371/journal.pone.0086923)

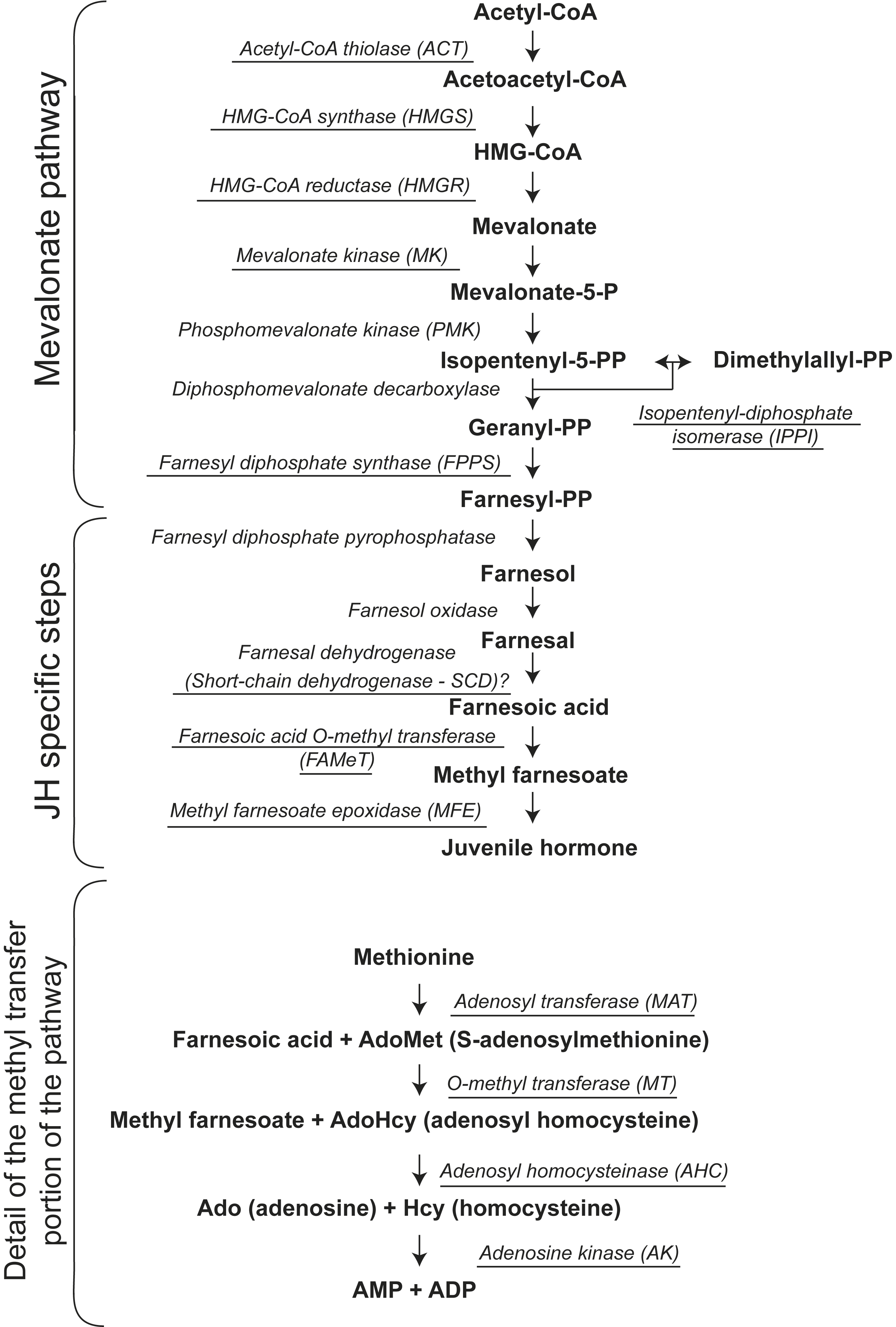

Supplement: Figure S1 — JH-III biosynthetic pathway (modified from Bellés et al. 2005 Annu Rev Entomol 50: 181–99, and Noriega et al. 2006 Insect Biochem Mol Biol 36:11 366–374). The underlined enzymes correspond to the genes whose expression is shown in Figure 1B–E . (TIF) [file pone.0086923.s001.tif]
